# Microbial control and a simple regenerative endodontic protocol achieve durable apical healing and long-term root development: A 10-year case report

**DOI:** 10.4317/jced.63482

**Published:** 2026-02-26

**Authors:** Mario Andrés Cumplido-Mendoza, Eliana Pineda-Vélez, Anny Marcela Vivares-Builes, Carlos M. Ardila

**Affiliations:** 1 Professor Basic Sciences Department, Faculty of Dentistry, Universidad de Antioquia U de A, Medellín 050010, Colombia; 2 Professor Basic Sciences Department, Biomedical Stomatology Research Group, Faculty of Dentistry, Universidad de Antioquia U de A, Medellín 050010, Colombia; 3 Professor Institución Universitaria Visión de las Américas, Medellín 050040, Colombia; 4 PhD. Professor Department of Periodontics, Saveetha Dental College, and Hospitals, Saveetha Institute of Medical and Technical Sciences, Saveetha University, Chennai 600077, India

## Abstract

Regenerative endodontics offers a biologically driven alternative to apexification for immature teeth with necrotic pulps and apical periodontitis. We report the management of an 11-year-old girl with a history of dentoalveolar trauma and an immature maxillary left central incisor (tooth 21) presenting with gray discoloration, negative sensibility, and a defined apical radiolucency (Nolla stage 9). Treatment followed a conservative, accessible protocol: disinfection with 5.25% sodium hypochlorite, placement of triple-antibiotic paste (ciprofloxacin, metronidazole, amoxicillin), and, at two weeks, irrigation with 17% EDTA, apical bleeding induction by controlled over-instrumentation, placement of a collagen matrix, and sealing with white mineral trioxide aggregate under a definitive composite restoration. A separate endodontic treatment was later performed on tooth 11 for mastication pain unrelated to the regenerative site. Long-term follow-up showed progressive and stable tissue regeneration. At 4 years, periapical radiography revealed early hard-tissue deposition in the middle and apical thirds and a slight increase in root length. At 8 years, marked root elongation and dentinal wall thickening were evident, improving structural prognosis. Cone-beam CT at 10 years confirmed complete apical closure, absence of periapical pathology, and substantial thickening of canal walls. Clinically, periodontal health remained intact and the tooth recovered a positive response to cold testing, consistent with true pulpal regeneration. This case demonstrates that meticulous microbial control combined with a simple, cost-conscious regenerative protocol may be associated with durable apical healing, continued root development, and functional recovery in an immature traumatized incisor. Although this is a single case and the findings are not generalizable, the sustained radiographic and clinical outcomes over a decade suggest that regenerative endodontics can be a feasible option in selected comparable cases when performed under appropriate clinical conditions.

## Introduction

The exposure of dentin to the oral cavity allows dentinal tubules to serve as an important pathway for bacterial penetration. When this is combined with evident lesions that compromise the pulp tissue (1), it leads to the development of apical periodontitis characterized by microbial organization in biofilms and predominance of anaerobic bacteria in extraradicular infections, as observed in apical abscesses (2). Therefore, the objective of this case report is to describe a simplified and widely accessible regenerative endodontic protocol for an immature traumatized incisor with apical periodontitis, and to highlight its novelty through long-term clinical and radiographic follow-up over 10 years, demonstrating durable apical healing and continued root development. Apical periodontitis is mediated by bacterial virulence factors, which may include structural components or metabolic products. In symptomatic periodontitis, there is a greater association with highly virulent species and planktonic microorganisms that display rapid growth and motility. In contrast, asymptomatic infections are more frequently associated with less virulent bacteria that exert a constant but persistent effect (3). During endodontic treatment, different shaping and cleaning strategies are employed to eliminate microorganisms using a variety of instruments. However, the chemical component provided by irrigants is fundamental to this process, with sodium hypochlorite being the primary agent. Clinical protocols also recommend complementary irrigants such as chlorhexidine, to enhance antimicrobial activity, and EDTA, to remove the smear layer generated during canal instrumentation (1 - 3). One of the greatest anatomical challenges arises in cases of pulp necrosis with apical periodontitis in immature teeth with open apices, which typically occur in very young patients. This condition represents a complex pathology with an unfavorable prognosis for long-term tooth preservation and additional consequences for maxillofacial development and dental esthetics (2 , 3). This pathology appears in teeth that have not yet completed root development and suffer pulp necrosis and apical periodontitis due to dental caries or dentoalveolar trauma. For many years, treatment relied on intracanal medication with calcium hydroxide. However, this approach frequently resulted in therapeutic failure due to the prolonged time required and the unpredictable formation of a reliable apical barrier. Later, the use of mineral trioxide aggregate (MTA) was introduced for creating an apical plug, which successfully resolved apical lesions but did not ensure long-term tooth survival (1 - 3). Consequently, current research focuses on alternative strategies, shifting from apexification with MTA to regenerative endodontic techniques. These approaches aim to overcome the limitations of achieving root lengthening and dentinal wall thickening (1 , 3). Treating immature teeth with open apices presents additional anatomical difficulties for canal cleaning, as proper mechanical shaping is not feasible. Furthermore, irrigants cannot be used at effective concentrations due to the risk of damaging periapical tissues, given the absence of an apical stop or barrier. A single-visit treatment is not always sufficient to achieve clinical success; therefore, intracanal medicaments such as calcium hydroxide and polyantibiotic pastes are commonly employed as adjuncts in the disinfection process (1 - 3). Current regenerative treatment protocols aim to, once microbial control is achieved, activate the regenerative triad of scaffold, stem cells, and signaling factors. This procedure involves thorough cleaning and disinfection, followed by apical stimulation to induce bleeding and clot formation. Platelet-based adjuncts such as platelet-rich fibrin may also be incorporated. Together with available growth factors and stem cells derived from the apical papilla, periodontal ligament, or bone, these components promote the formation of mineralized tissue within the canal (4). The purpose of the present case report is to describe the use of a simplified and widely accessible regenerative endodontic protocol in an immature traumatized tooth with apical periodontitis, and to report the long-term clinical and radiographic outcomes observed over a 10-year follow-up, including apical healing and continued root development.

## Case Report

**Figure 1:**
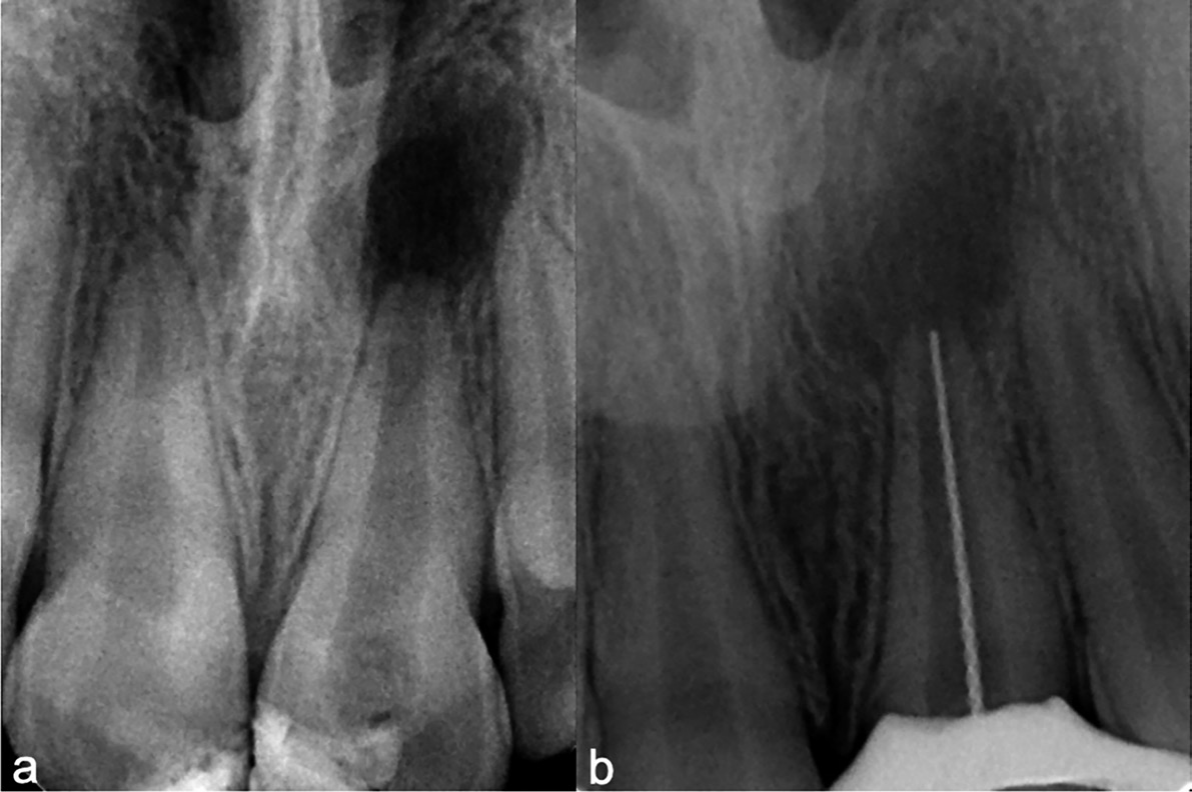
Baseline and intraoperative periapical radiographs of tooth 21. (a) Preoperative periapical radiograph showing an immature apex (Nolla stage 9) and a well-defined apical radiolucency consistent with asymptomatic apical periodontitis in an 11-year-old patient with a history of dental trauma. (b) Working length determination radiograph (21 mm) using a #40 K-file during canal disinfection and preparation prior to regenerative endodontic therapy.

An 11-year-old female patient attended the School of Dentistry at the University of Antioquia, Medellín, Colombia. She had no history of systemic diseases or medication use. The patient came with her mother, both of whom received detailed information about the procedure. They accepted the treatment plan and signed the institutional informed consent form. The patient reported a history of dentoalveolar trauma due to a fall from her own height, with an evolution of two years, for which she had not previously sought treatment. The chief complaint was: "the tooth has a different color compared to the other teeth." At the clinical examination, tooth 21 presented with a grayish discoloration, a negative response to pulp sensibility tests, and radiographically a defined apical radiolucent area with an immature apex, classified as Nolla stage 9. The diagnosis was previously initiated therapy and asymptomatic apical periodontitis (Fig. 1a). A regenerative endodontic procedure was planned for tooth 21 (Fig. 1a).

[caption id="attachment_2115" align="alignnone" width="300"]1 Screenshot[/caption]

**Figure 2:**
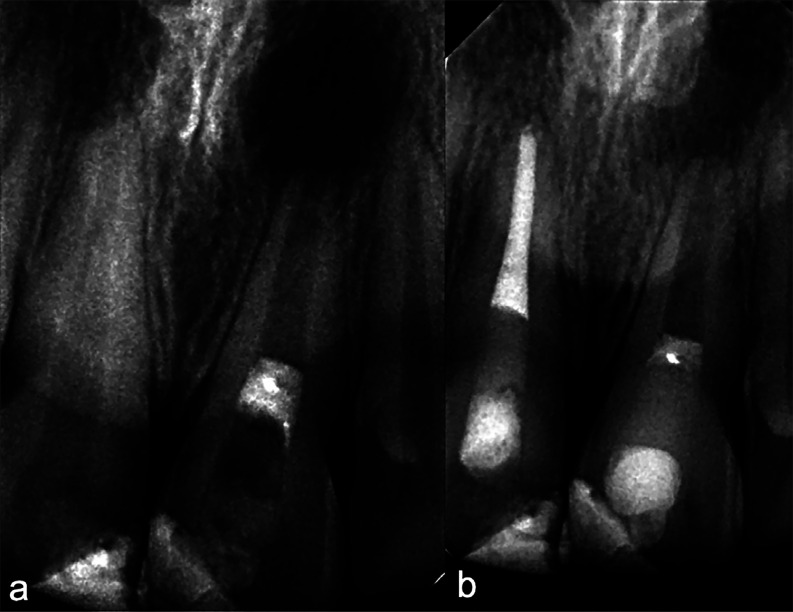
Postoperative and long-term periapical radiographic follow-up of tooth 21 after regenerative endodontic therapy. (a) Postoperative radiograph showing placement of the cervical MTA barrier over the collagen matrix/blood clot scaffold. (b) Four-year follow-up periapical radiograph showing resolution of the apical radiolucency and evidence of continued root development, including increased root length and hard tissue deposition in the middle and apical thirds.

The procedure began with intraoral disinfection using 0.12% chlorhexidine mouthwash for one minute, followed by topical anesthesia and infiltration with 2% lidocaine with epinephrine 1:80,000, one carpule. Absolute isolation was achieved with a rubber dam and Hu-Friedy® clamp No. 6. Access cavity preparation was performed with a round bur No. 6, and irrigation with a Monoject syringe and a 27G needle using 5 ml of 5.25% sodium hypochlorite (NaOCl). Working length was established at 21 mm with a K-file #40 (Dentsply® Maillefer®) and verified radiographically (Fig. 1b). Irrigation was continued with 20 ml of 5.25% NaOCl, followed by a rinse with distilled water and drying with sterile paper points. Finally, triple antibiotic paste (TAP) composed of metronidazole, ciprofloxacin, and amoxicillin was placed. Tablets of ciprofloxacin 500 mg, metronidazole 500 mg, and amoxicillin 500 mg were crushed in a sterile mortar to obtain a fine powder, mixed in equal proportions (1:1:1), and combined with sterile saline as a vehicle. The paste was introduced into the canal with a Lentulo spiral, filling it up to 3 mm short of the pulp chamber. Excess material was removed, a sterile cotton pellet was placed, and the access was temporarily sealed with Vitremer® glass ionomer (3M ESPE®). No systemic antibiotics or analgesics were prescribed, as the patient was asymptomatic. Two weeks later, the patient returned for the continuation of therapy on tooth 21. In this appointment, intraoral disinfection with 0.12% chlorhexidine mouthwash for one minute was performed, followed by topical anesthesia with Roxicaina® and infiltration with lidocaine without epinephrine, one carpule. Absolute isolation was achieved with a rubber dam and Hu-Friedy® clamp No. 6. The temporary restoration was removed, and the canal was rinsed with sterile distilled water to eliminate TAP. Irrigation with 17% EDTA (Coltene®) was performed for one minute, after which a K-file #40 (Dentsply® Maillefer®) was used to induce bleeding by overinstrumentation approximately 4 mm beyond the apical foramen. Bleeding was allowed to fill the canal until 2 mm below the pulp chamber. At this point, a collagen matrix (Collaplug® Zimmer Biomet®) was placed, followed by 3 mm of white mineral trioxide aggregate (MTA) PRO ROOT®. A moist sterile cotton pellet was placed to allow cement setting, and the tooth was temporarily sealed with Vitremer® glass ionomer (3M ESPE®). Three days later, the temporary restoration and cotton pellet were removed, the stability of the MTA was verified, and the tooth was permanently restored with light-cured composite resin shade A2 Z100® (3M ESPE®). One year later, the patient presented complaining of pain on mastication, pointing to tooth 11. The tooth tested negative to pulp sensibility, had a grade 3 percussion response, probing depths of 2 mm on all surfaces, and radiographically showed no apical lesion but a slight widening of the periodontal ligament space. Endodontic treatment was initiated on tooth 11. Local anesthesia was achieved by infiltration of one carpule of 2% lidocaine with 1:80,000 epinephrine, ensuring adequate analgesia and hemostasis. Absolute isolation was established with a rubber dam and Hu-Friedy® clamp No. 6. Access cavity preparation was performed, and the canal was explored with a manual #15 K-file (Dentsply® Maillefer®). Working length was determined at 22 mm with an electronic apex locator (Root ZX® J. Morita®) and confirmed with a K-file #25 (Dentsply® Maillefer®). Biomechanical preparation was performed with Reciproc® R50 (VDW) following manufacturer's recommendations while maintaining working length. During instrumentation, the canal was irrigated with 20 ml of 5.25% NaOCl using a Monoject syringe and a 27G needle. A final rinse with sterile saline was performed, and the canal was dried with sterile paper points. Calcium hydroxide was placed as intracanal medication, and the access cavity was temporarily sealed with Vitremer® glass ionomer (3M ESPE®). Treatment was completed in two visits, two weeks apart. At the second visit, the temporary restoration was removed, the canal was irrigated with 10 ml of 5.25% NaOCl, rinsed, dried, irrigated with 17% EDTA (Coltene®), ultrasonically activated, rinsed again, dried, and obturated with gutta-percha and TOPSEAL® sealer (Dentsply®). The excess was removed, and the access cavity was sealed with Vitremer® glass ionomer (3M ESPE®) and restored with light-cured composite resin shade A2 Z100® (3M ESPE®). Although lower NaOCl concentrations are often recommended in regenerative endodontic protocols to reduce cytotoxicity, 5.25% NaOCl was selected in this case due to the presence of apical periodontitis and the need for enhanced disinfection. Irrigation was performed gently using a Monoject syringe and a 27G needle, with strict attention to avoid apical extrusion and minimize potential adverse effects on apical stem cells. Radiographic follow-ups were performed at 4 and 8 years, and cone-beam computed tomography (CBCT) at 10 years. At the 4-year periapical radiograph (Fig. 2a,b), a slight increase in root length of tooth 21 and hard tissue deposition in the middle and apical thirds were observed.

[caption id="attachment_2116" align="alignnone" width="300"]2 Screenshot[/caption]

**Figure 3:**
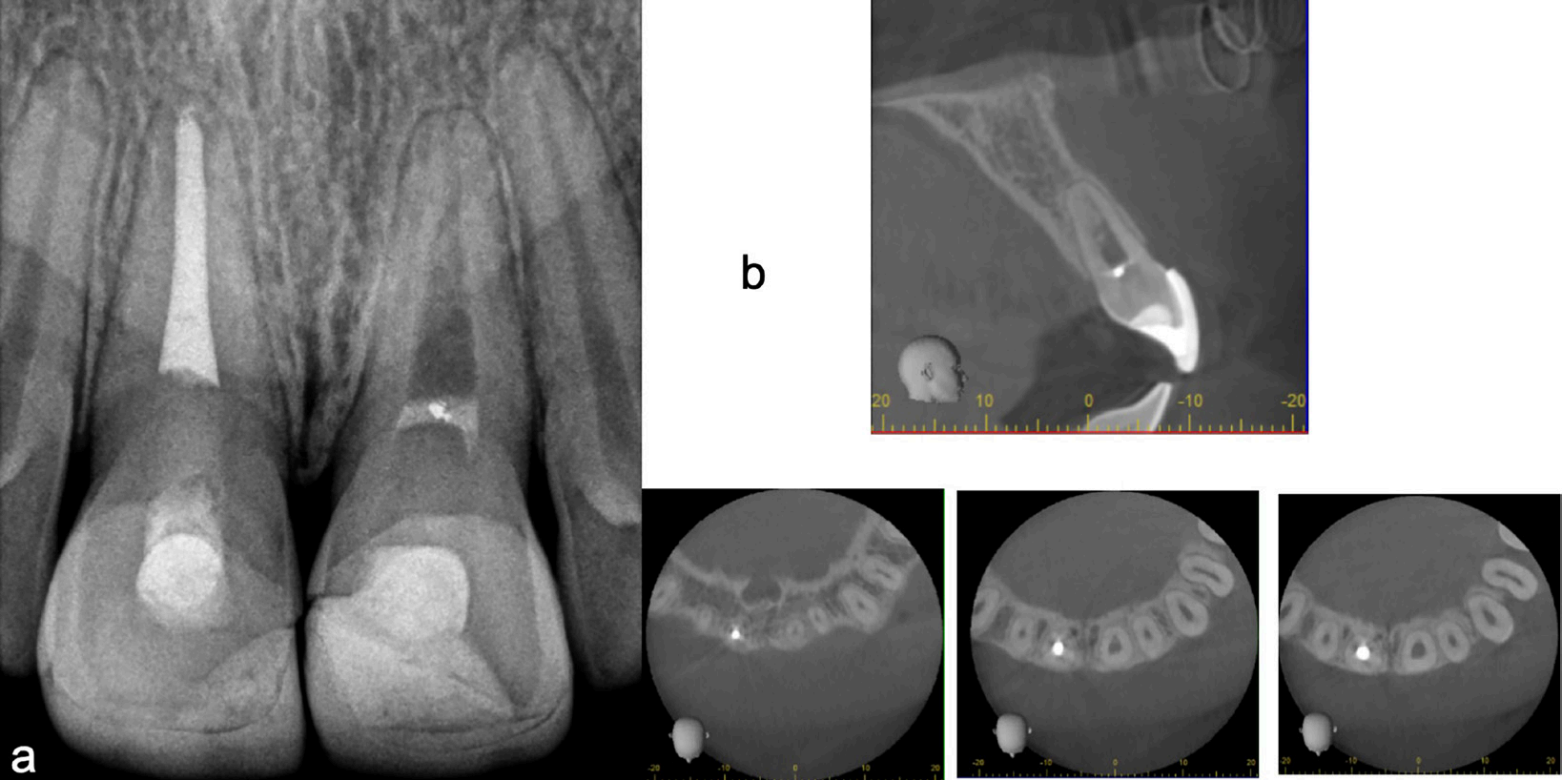
Late radiographic follow-up demonstrating continued root maturation and apical healing of tooth 21. (a) Eight-year follow-up periapical radiograph showing marked increase in root length and thickening of dentinal walls, consistent with progressive root maturation. (b) Ten-year follow-up CBCT confirming apical closure, absence of periapical lesions, and increased thickness of the root canal walls, supporting long-term apical healing and root development.

At the 8-year follow-up (Fig. 3a), a considerable increase in root length and thickening of the dentinal walls were noted, improving the prognosis of the tooth. Two years later, CBCT demonstrated complete apical closure, absence of apical lesions, and a substantial increase in root canal wall thickness (Fig. 3b). This case is a descriptive single-patient case report. Therefore, sample size calculation, operator blinding, and statistical analysis are not applicable. All clinical procedures, follow-up visits, and outcome assessments were performed by the same operator.

[caption id="attachment_2117" align="alignnone" width="300"]3 Screenshot[/caption]

## Discussion

The objective of this case report was to describe the clinical management of an immature traumatized maxillary incisor with asymptomatic apical periodontitis using a simplified and widely accessible regenerative endodontic protocol, and to document the long-term outcomes. Clinically, the tooth remained functional and asymptomatic, while radiographic follow-up demonstrated progressive resolution of the apical lesion and continued root maturation. At 4 years, periapical radiography showed apical healing with evidence of increased root length and hard tissue deposition; at 8 years, further root elongation and dentinal wall thickening were evident; and at 10 years, CBCT confirmed apical closure, absence of periapical pathology, and increased thickness of the root canal walls. These findings provide the basis for discussing biological mechanisms and clinical considerations underlying regenerative endodontic outcomes in immature teeth. Apical periodontitis is primarily driven by microbial infection of the root canal system, and persistent inflammation contributes to lesion maintenance (5). Accordingly, effective disinfection remains central to both conventional and regenerative endodontic procedures. Canal irrigation supports antimicrobial control, tissue dissolution, smear-layer removal, and canal lubrication (6). In immature teeth, achieving adequate disinfection is particularly challenging due to open apices, thin dentinal walls, and the risk of apical extrusion or tissue damage. Optimal treatment aims to reduce the intracanal bacterial load as much as possible to control apical inflammation and promote healing. Endodontic therapy is performed under aseptic conditions to prevent bacterial recontamination, combining mechanical and chemical procedures. Mechanical instrumentation involves various shaping systems with files differing in alloy and taper, complemented by chemical antimicrobial irrigants in different concentrations, which are activated within the canal and may also remain as intracanal medication between appointments to impact microbial communities (biofilms). Irrigation during preparation assists in disinfection, dissolution of soft tissue, removal of the smear layer, and lubrication of instruments (6). In regenerative endodontic procedures, NaOCl concentration is clinically relevant because higher concentrations may be cytotoxic to apical stem cells. Lower concentrations are commonly recommended; however, in infected cases, stronger disinfection strategies may be required. In the present case, the use of 5.25% NaOCl was balanced with conservative irrigation to minimize extrusion, followed by EDTA irrigation prior to induction of bleeding. Thus, the primary goal of endodontics-to eliminate as many microorganisms as possible from the root canal system and to create conditions that limit the survival and regrowth of residual bacteria-becomes even more complex in immature teeth. These teeth are fragile, difficult to obturate tridimensionally, and at risk of overfilling and apical tissue damage. For this reason, the American Association of Endodontists introduced the concept of "endodontic regeneration" in 2007 (7 , 8), based on tissue engineering principles of the regenerative triad: stem cells, a biomimetic scaffold, and bioactive growth factors within the root canal space, with the goal of improving prognosis and survival of teeth compromised by caries or trauma (7). Pulpal regeneration is a biologically based treatment for immature teeth with necrotic pulp that, unlike apexification, may allow continued root development with dentinal wall thickening and root elongation in selected cases. This therapy involves a scaffold derived from a blood clot, signaling growth factors released from dentin, and stem cells originating from tissues adjacent to the apex (9). Various intracanal medicaments have been used for cleaning immature teeth with open apices, most commonly calcium hydroxide and polyantibiotic pastes. When complete disinfection (absence of pain or inflammation) is not achieved, polyantibiotic pastes composed of ciprofloxacin, metronidazole, and minocycline are used (10). The clinical use of triple antibiotic paste in regeneration is recommended in a 1:1:1 ratio (ciprofloxacin, metronidazole, and minocycline) at a final concentration of 1-5 mg/ml to minimize potential adverse effects on periodontal ligament stem cells. Research has explored different types of hydrogels as vehicles for intracanal medicaments, focusing on inflammation control, blood supply promotion, extracellular matrix mimicry, controlled signaling molecule release, modulation of stem cell behavior, and appropriate physicochemical properties. Among the most widely used are collagen, chitosan, and hyaluronic acid. Hydrogels have gained popularity as scaffolding materials and can be loaded with drugs or bioactive compounds (11). An ideal scaffold has yet to be identified. Ongoing studies aim to construct and combine scaffolds or load them with biomolecules, peptides, antibiotics, and growth factors. Although blood clot (BC), platelet-rich plasma (PRP), platelet-rich fibrin (PRF), and clot-collagen membrane combinations are currently used in the clinic, results are inconsistent and new alternatives continue to emerge. Additionally, collagen/gelatin hydrogels enriched with fibronectin (FN) may activate apical papilla cells by promoting migration, viability, adhesion, spreading, and gene expression (12). It is well established that mesenchymal stem cells (MSCs) persist in adults and participate in tissue turnover (13). Regeneration occurs via chemotaxis of these cells to the injured site through signaling molecules. Successful outcomes are thought to depend on the recruitment and differentiation of stem cells, as well as the presence of an appropriate scaffold. Growth factors, polypeptides, and proteins direct a broad spectrum of cellular activities, including migration, proliferation, differentiation, and maturation. Endogenous growth factors present in dentin walls can be released after disinfection, with more than 300 proteins identified in human dentin, most of them involved in cell growth, communication, metabolism, and immunity. Platelet-derived growth factors, vascular endothelial growth factor (VEGF), and fibroblast growth factors are also essential in promoting stem cell migration, proliferation, and differentiation (14). Some authors have investigated autologous pulp stem cell transplantation for regenerating the pulp-root complex. In such cases, mineralized tissue formation at the apex and recovery of vitality responses have been documented, even in mature teeth with apical periodontitis, demonstrating the biological feasibility of cellular therapy. However, this approach involves greater technical complexity, longer intervention times, and morbidity at the donor site, limiting its routine application, in addition to ethical concerns. More recently, strategies using undifferentiated mesenchymal stem cells (MSCs) without in vitro differentiation protocols have been reported. Direct administration of MSCs into the pulp space has led to root regeneration within as little as four months of follow-up, even in teeth previously treated with conventional endodontics. Despite these promising findings, controlled studies, standardized protocols for cell handling, and cost-effectiveness analyses are needed before recommending widespread use (15). In the last decade, there has been a steady increase in published case reports on pulp regeneration in immature teeth, averaging twenty-five reports annually. To progress toward a predictable and accessible regenerative treatment model, it is essential to standardize disinfection and scaffold placement methods, conduct randomized clinical trials comparing different techniques, and rigorously assess cost-benefit relationships across clinical settings. In this case, regenerative endodontic therapy demonstrated resolution of the apical lesion, root lengthening, apical closure, and thickening of root canal walls. Clinical periodontal evaluation and pulp sensibility tests showed a positive response to cold testing and integrity of periodontal tissues, supporting the evidence of true pulp regeneration. Regeneration is not yet a common clinical treatment, and protocols vary widely, reflecting the heterogeneity of published data and multifactorial determinants of success. Nonetheless, this case demonstrates that careful microbial control combined with a simple protocol can achieve significant outcomes that may be applied routinely in similar cases. Several limitations inherent to this report should be acknowledged. As a single-case report, the present findings cannot be generalized and do not establish the predictability of regenerative endodontic therapy. Moreover, histological confirmation was not available; therefore, true pulp tissue regeneration cannot be verified. In addition, a positive sensibility test should be interpreted cautiously because sensibility responses may reflect neural reinnervation or the presence of vital tissue rather than full regeneration of the native pulp-dentin complex.

## Conclusions

This case report describes the use of regenerative endodontic therapy based on tissue-engineering principles (the regenerative triad: stem cells, a scaffold, and bioactive growth factors) in an immature tooth affected by trauma-associated apical periodontitis. The favorable clinical and radiographic outcomes observed over long-term follow-up suggest that, in selected cases and under appropriate clinical conditions, this approach may contribute to apical healing and continued root development. However, as these findings are derived from a single observational case, they should be interpreted with caution and cannot be generalized without further controlled clinical evidence.

## Data Availability

Records were obtained from the included investigations.

## References

[1] Tong HJ, Seremidi K, Stratigaki E, Kloukos D, Duggal M, Gizani S (2022). Deep dentine caries management of immature permanent posterior teeth with vital pulp: a systematic review and meta-analysis. J Dent.

[2] Arias Z, Nizami MZI, Chen X, Chai X, Xu B, Kuang C (2023). Recent Advances in Apical Periodontitis Treatment: A Narrative Review. Bioengineering (Basel).

[3] Neelakantan P, Herrera DR, Pecorari VGA, Gomes BPFA (2019). Endotoxin levels after chemomechanical preparation of root canals with sodium hypochlorite or chlorhexidine: a systematic review of clinical trials and meta-analysis. Int Endod J.

[4] Baumgartner JC, Cuenin PR (1992). Efficacy of several concentrations of sodium hypochlorite for root canal irrigation. J Endod.

[5] Meirinhos J, Martins JNR, Pereira B, Baruwa A, Gouveia J, Quaresma SA (2020). Prevalence of apical periodontitis and its association with previous root canal treatment, root canal filling length and type of coronal restoration - a cross-sectional study. Int Endod J.

[6] Rôças IN, Ricucci D, Hülsmann M (2014). Causes and management of post-treatment apical periodontitis. Br Dent J.

[7] Murray PE, Garcia-Godoy F, Hargreaves KM (2007). Regenerative endodontics: a review of current status and a call for action. J Endod.

[8] Law AS (2013). Considerations for regeneration procedures. J Endod.

[9] Schmalz G, Widbiller M, Galler KM (2020). Clinical perspectives of pulp regeneration. J Endod.

[10] Hegde VR, Jain A, Patekar SB (2023). Comparative evaluation of calcium hydroxide and other intracanal medicaments on postoperative pain in patients undergoing endodontic treatment: a systematic review and meta-analysis. J Conserv Dent.

[11] Samiei M, Alipour M, Khezri K, Saadat YR, Forouhandeh H, Abdolahinia ED (2022). Application of Collagen and Mesenchymal Stem Cells in Regenerative Dentistry. Curr Stem Cell Res Ther.

[12] Leite ML, Soares DG, Anovazzi G, Anselmi C, Hebling J, de Souza Costa CA (2021). Fibronectin-loaded Collagen/Gelatin Hydrogel Is a Potent Signaling Biomaterial for Dental Pulp Regeneration. J Endod.

[13] Sawada Y, Honda T, Hanakawa S, Nakamizo S, Murata T, Ueharaguchi-Tanada Y (2015). Resolvin E1 inhibits dendritic cell migration in the skin and attenuates contact hypersensitivity responses. J Exp Med.

[14] Yan H, De Deus G, Kristoffersen IM, Wiig E, Reseland JE, Johnsen GF (2023). Regenerative Endodontics by Cell Homing: A Review of Recent Clinical trials. J Endod.

[15] Gomez-Sosa JF, Diaz-Solano D, Wittig O, Cardier JE (2022). Dental Pulp Regeneration Induced by Allogenic Mesenchymal Stromal Cell Transplantation in a Mature Tooth: A Case Report. J Endod.

